# Symptomatic Mixed Cryoglobulinemia during HIV Primary Infection: A Case Report

**DOI:** 10.1155/2011/525841

**Published:** 2011-11-20

**Authors:** Philippe Genet, Laurence Courdavault, Bouchra Wifaq, Juliette Gerbe

**Affiliations:** ^1^Department of Hematology, Hôpital Victor-Dupouy, 95100 Argenteuil, France; ^2^Department of Virology, Hôpital Victor-Dupouy, 95100 Argenteuil, France

## Abstract

We report a patient who developed during HIV primary infection a symptomatic mixed cryoglobulinemia. The patient suffered from arthralgias, vascular purpura of the legs, and proteinuria. Cryoglobulinemia progressively disappeared in several months after HAART.

Cryoglobulinemia is a common finding during the course of HIV disease, especially for patients coinfected with HCV. Nevertheless, cryoglobulinemia is rarely symptomatic in HIV-monoinfected patients. We present herein a patient with symptomatic cryoglobulinemia leading to the discovery of HIV primary infection.

A 31-year-old man was referred to us for a symptomatology of 2-week duration. Past medical history was unremarkable. The patient reported unprotected receptive oral sex one month earlier with a male whose status for HIV was unknown. The patient complained from abdominal pain, arthralgia of ankles and wrists, and purpura of legs.

On examination, an extensive vascular purpura of the legs was present without other abnormalities. Biopsy of the skin revealed signs of vasculitis. Standard biological exams were normal, except for a mild proteinuria (0.5 g/L). Creatinemia was normal (0.9 mg/dL). Complement was normal. Anti-DNA antibodies were negative. A mixed cryoglobulinemia was detected in the serum. HCV and HBV serology were negative. HCV serology was done one year later and remained negative.

HIV testing was positive on ELISA while an incomplete profile was found on western blot (gp120 +/−, gp41++++, p31−, p24+). p24 antigenemia was positive (28 pg/mL). HIV viral load (VL) was 4.7 log copies/mL. CD4 cells count was 213/mm^3^. Antiretroviral therapy (tenofovir, emtricitabine, and r/atazanavir) was given. After 3 months of treatment, VL was <50 copies/mL and CD4 cells count was 924/mm^3^. Western blot was repeated and was subsequently positive for all bands tested. During this period, symptoms progressively decreased with a complete resolution after 3 months. Cryoglobulinemia became negative after 1 year of treatment. After a followup of 3 years, the patient remained asymptomatic with CD4 cells count always above 500/mm^3^ and VL <50 copies/mL.

Prevalence of cryoglobulinemia is high in HIV-infected patients. Scotto et al. [1] found a prevalence of 6% for HIV-monoinfected patients and 14.2% for HIV-HCV-coinfected patients. In spite of this high prevalence, cryoglobulinemia is rarely symptomatic [2] even if rare neurological, dermatological, renal, or rheumatological complications have been described [3]. These complications occurred usually during chronic phase of HIV infection. To our knowledge, it has been not reported during acute primary infection. At presentation, our patient clearly suffered from symptomatic cryoglobulinemia. Clinical and anatomopathological findings were consistent with this hypothesis ruling out an incidental discovery of a cryoglobulinemia during HIV infection. The simultaneous occurrence of symptomatic cryoglobulinemia and HIV primary infection argued in favour of a role of HIV in the appearance of cryoglobulinemia. The impact of HAART on disappearance of cryoglobulinemia in our observation is difficult to establish.

Nevertheless, we cannot rule out a positive effect given that, in epidemiologic studies, prevalence of cryoglobulinemia in HIV-infected patients has decreased in HAART era [4].
